# Insight into the Mechanism of d-Glucose Accelerated Exchange in GLUT1 from Molecular Dynamics Simulations

**DOI:** 10.1021/acs.biochem.4c00502

**Published:** 2025-01-28

**Authors:** Carmen Domene, Brian Wiley, Saul Gonzalez-Resines, Richard J. Naftalin

**Affiliations:** † Department of Chemistry, University of Bath, Claverton Down, Bath BA2 7AY, United Kingdom; ‡ BHF Centre of Research Excellence, School of Medicine and Life Sciences, King’s College London, London SE1 9NH, United Kingdom

## Abstract

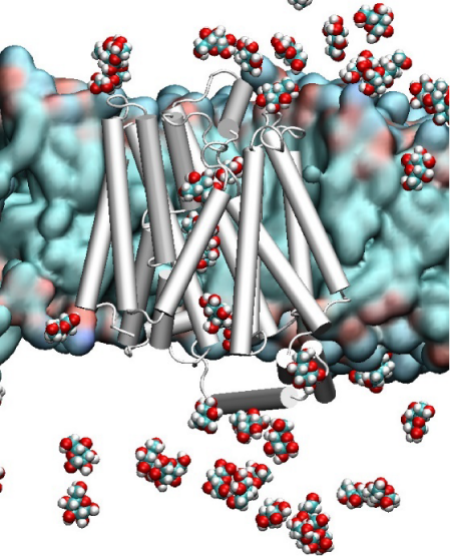

Transmembrane glucose transport, facilitated by glucose transporters (GLUTs), is commonly understood through the simple mobile carrier model (SMCM), which suggests that the central binding site alternates exposure between the inside and outside of the cell, facilitating glucose exchange. An alternative “multisite model” posits that glucose transport is a stochastic diffusion process between ligand-operated gates within the transporter’s central channel. This study aims to test these models by conducting atomistic molecular dynamics simulations of multiple glucose molecules docked along the central cleft of GLUT1 at temperatures both above and below the lipid bilayer melting point. Our results show that glucose exchanges occur on a nanosecond time-scale as glucopyranose rings slide past each other within the channel cavities, with minimal protein conformational movement. While bilayer gelation slows net glucose transit, the frequency of positional exchanges remains consistent across both temperatures. This supports the observation that glucose exchange at 0 °C is much faster than net flux, aligning with experimental data that show approximately 100 times the rate of exchange flux relative to net flux at 0 °C compared to 37 °C.

## Introduction

Accelerated d-glucose exchange is a phenomenon observed in several members of the passive d-glucose transporter group from the facilitated glucose transporter member 1 (SLC2A class 1) family, also known as GLUTs. This trans-acceleration effect occurs in GLUT/SLC2A transporters 1 and 3^1^ but not in GLUTs 2^2,3^ or GLUT 4.^4,5^ Understanding this phenomenon is crucial for studying the mechanisms of d-glucose transport, particularly in the context of its foundational role in the development of glucose transport theory in the mid-twentieth century.

Hypothetical explanations of d-glucose transport mechanisms were proposed long before any structural knowledge of transporters was available. Surprisingly, despite significant advances in understanding the structures of glucose transporters, early views on the mechanism of d-glucose exchange transport have remained substantially unchanged and virtually unchallenged. This paper aims to discriminate between current transport models by analyzing atomistic d-glucose exchange trajectories within GLUT1 using atomistic molecular dynamics simulations with protocols designed to optimize the occurrence of exchange events. “Saturating docking” simulations were employed, in which multiple ligands are initially placed within the GLUT1 transporter along the length of the central cleft, between the first (transmembranes, TMs 1–6) and second (TMs 7–12) transmembrane helical sextets. These protocols are compared with others where the external solutions are “flooded” additionally with high glucose concentrations, equivalent to 50 mM, to simulate conditions at the maximal rate of equilibrium exchange.

**Scheme 1:**
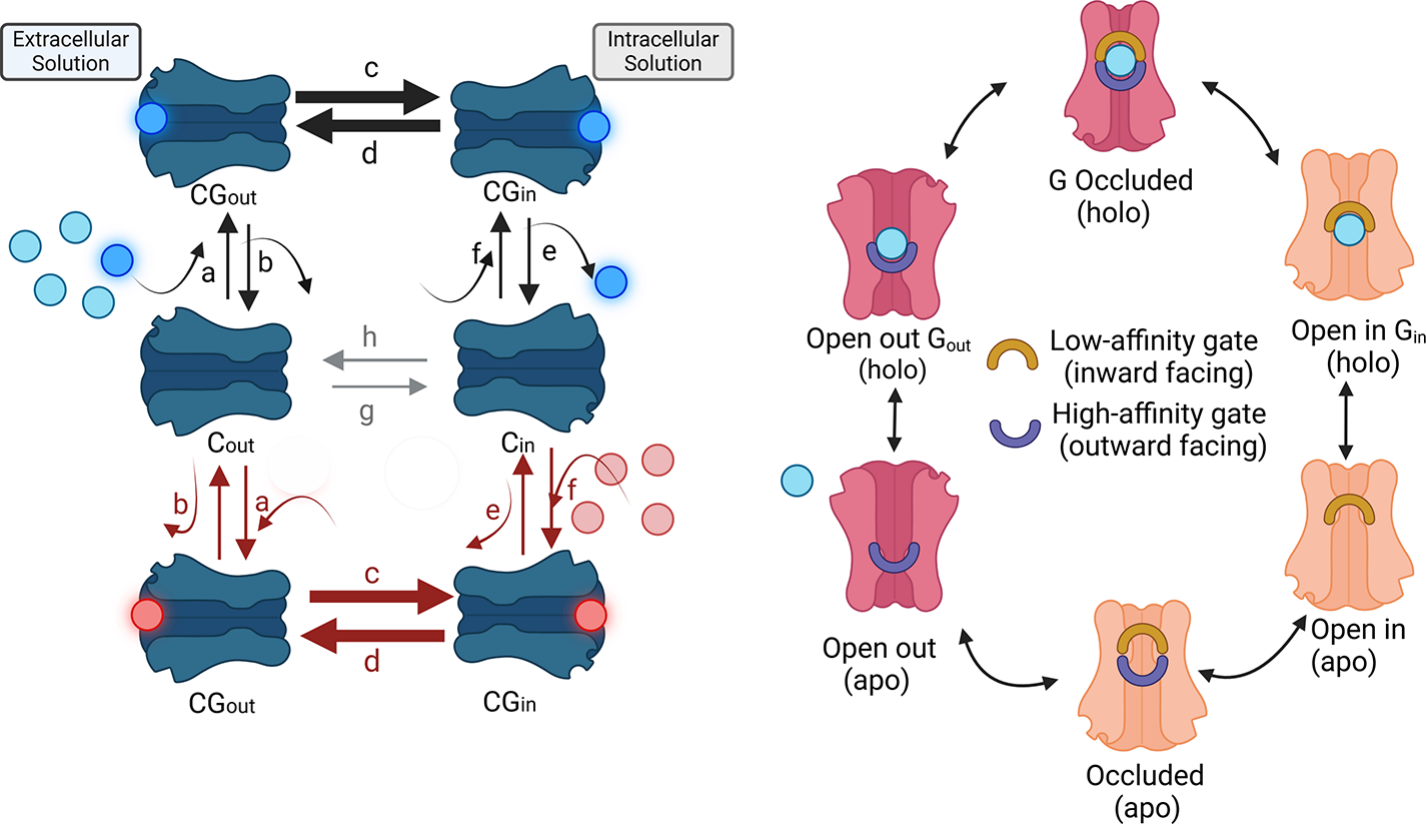
(a) Conventional Diagram of Simple Mobile Carrier Model Where Internal Glucose (Red) Exchanges Sequentially with External Glucose (Blue) (a) *C*_in_/*C*_out_ refers to unliganded carrier facing inwards or outwards and *CG*_in_ /*CG*_out_ to the liganded carriers. Rate coefficients a–h refer to the unidirectional rate constants of the networks. The inflow unidirectional rates of the external glucose are shown in the upper half of the diagram with clockwise directed red arrows and the outflow of internal glucose in the lower half with counterclockwise facing arrows. Thicker arrows represent transmembrane unidirectional glucose outflow and inflow and symbolise faster via the liganded holo branches. Net glucose transit requires a complete cycle around either the upper cycle for influx (a → c → e → h) or for the cycle for net efflux (f → d → b → g). Net flux is slower than exchange. Exchange requires sequential movements of glucose via the holo branches e.g., a → c → e → f → h → b. Net glucose transit requires a complete cycle around either the upper cycle for influx (black a → black c → black e → gray h) or for the lower cycle for net efflux (red f → red d → red b → gray g). All the individual steps are reversible. (b) A modification of the network diagram depicted in panel (a) showing the conventional circulating mobile carrier model (SMCM) with outside high affinity gate and inside low affinity gate and occluded central site. Double-gated carrier cycle incorporating large scale rigid transmembrane helical conformational changes with synchronized gate openings and closures. This is like the conventional alternating access “Jardetzky model”^5^ but, it incorporates an occluded intermediate state. Net flux requires a complete circuit whereas exchange only requires sequential transits via the top and lowest branches in (a) and omitting the central unliganded “apo” branch. The blue glucose ligand binds to the high affinity (blue) outward facing gated site (open out GOout) holo, and then becomes occluded by closure of the second low affinity inward facing gate. The high affinity gate opens releasing glucose from the low affinity gate to the inside solution, leaving the central binding site empty (apo state). The cycle continues via the apo occluded state to open outward apo state when the cycle can restart. Net flux requires a complete circuit whereas exchange only requires sequential transits via the top branch and lowest branches. Double gated model with high affinity outside gate and low affinity inside gate and occluded central site in which the ligands are free to inter diffuse without significant frictional interference between the external and internal sites or without net energy transfer between the two interfacial sites, as is implicit in the SMCM model with asymmetric affinities.^41^ This scheme was created in Biorender (Domene, C. (2025) https://BioRender.com/d07b866).

Recent structural studies of d-glucose transport by GLUTs have focused primarily on how net transport correlates with protein conformational changes.^6−9^ These studies have been guided by the assumptions of the single-site alternating access transport model. This model’s main assumption is that there is a single high-affinity ligand binding site within the transporter, which alternates between exposing the ligand to the inside and outside bathing solutions at the cell membrane surfaces. The “simple mobile carrier model” (SMCM) implies a conformational change where the bound ligand complex sequentially reorients from one side to the other (see Scheme 1).^10^ A later modification of this ferry boat model incorporates a double gating sequence within the transit pathway that facilitates net transport while simultaneously enacting the other functional properties of GLUT1 hexose transport, namely, imposing ligand stereospecificity and saturability and preventing nonspecific ligand and water leakage (see Scheme 1). These two processes are often considered analogous to a reversible toggle-switch motion between inward-facing and outward-facing states.^5^

The SMCM for net transport implies a sequential cyclic flow in which ligand binding to the *cis* transport site cycles across the membrane in the liganded, or holo forms (Scheme 1a, rates c,d) to the *trans* side, where the ligand dissociates into the adjacent solution. In this context, the terms *cis* and *trans*, used by Lieb and Stein,^11−13^ refer to the *cis* side from which the ligand is transported, and the *trans* side to which the ligand is transported. To complete and reinitiate the cyclic net transport process, the unliganded, or apo, form of the transporter site must return from the *trans* to the *cis* side (Scheme 1a, rates g,h). If the transporter encounters and binds a ligand on the *trans* side after a *cis* to *trans* transport event, then the substitute ligand returns to the *cis* side via the liganded, or holo, form of the transport arm of the cycle (Scheme 1a, rates g,h). This latter process is regarded as the sequential mode of ligand exchange and is only detectable if the *trans* ligand is distinguishable from the *cis* ligand, i.e., carries a differentiating label.^11^ If the mobility of the holo-exchange branch is faster than that of the apo-exchange branch, then exchange ligand transport will appear to be faster than net flux, that is, accelerated. Exchange glucose transport, measured with radioisotopically labeled d-glucose, has been shown to be considerably faster than net transport.^14−18^

The closed sequence of the SMCM passive net flux cycle also implies intrinsic thermodynamic and kinetic constraints. The first of these is microscopic reversibility, whereby to achieve “detailed balancing” at equilibrium, the product of the clockwise rates around the complete cycle at equilibrium, as shown in Scheme 1, must equal the product of the counterclockwise rates.^19^ The Haldane relationship, derived for enzyme kinetic treatments of reversible reactions within a homogeneous solution, embodies these constraints and is also incorporated in Lieb and Stein’s rejection criteria.^12,13^ If observed transporter parameters fall outside the constraints of these rejection criteria, then the transporter mechanism cannot be classed as a single-site cyclic process and, therefore, does not conform with the SMCM.

The downhill or passive flow of d-glucose or other transported sugars, such as D-galactose or xylose, can generate a simultaneous transient uphill movement of labeled sugar. This phenomenon, termed counterflow,^14^ was demonstrated convincingly in erythrocytes by observing an uphill influx of a low concentration of radioactively labeled d-glucose.^15,20,21^ Counterflow has been proposed as qualitative evidence favoring d-glucose transport via a mobile carrier. However, repeated mathematical analyses of the integrated counterflow hexose transients, based on SMCM theory, have shown discrepancies with the observed trajectories.^20,22−25^

The d-glucose exchange process was originally conceptualized as operating like a swing door or ferryboat.^14^ Within the framework of the SMCM, when the inside solution contains d-glucose at saturating concentrations (≈50–100 mM), net d-glucose downhill efflux via the more rapidly moving arm of the mobile-carrier complex (Scheme 1a, rate d) results in the empty carrier component orienting in the outward-facing pose. This orientation permits higher maximal rates of d-glucose exchange inflow (via Scheme 1a, rate c), as relatively more sites are available for d-glucose inflow. This protocol is termed infinite-*trans* influx and is faster than when the d-glucose influx occurs in the absence of glucose from the inside solution.^26^ This latter process is termed the zero-*trans* net entry condition, as the cytosol is initially nominally d-glucose-free, and net d-glucose influx is supposedly rate-limited by the slow rate of return of the empty carrier (Scheme 1 rate h).

After the inflowing ligand reaches the inside compartment, if isotopically labeled d-glucose is present in the *trans* compartment, the return event will carry a labeled d-glucose ligand from the inside to the outside, which can be measured as exchange efflux. If no d-glucose binds on the *trans* side, the empty carrier will return to the external side more slowly, and the process will be registered as net d-glucose entry.^27^

Since the exchange process shares one of the two sequential transmembrane paths defining the net flux network, it is partially incorporated into the net flux mechanism, making ratios of the maximal rates of equilibrium exchange (*V*_ee_) and zero-*trans* net influx (*V*_oi_) to net zero-*trans* efflux (*V*_io_) predictable.^13,20,28−31^ The predicted ratio is
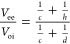
1where *V*_ee_ is the *V*_max_ of the equilibrium-exchange condition and *V*_oi_ is the *V*_max_ for zero-*trans* net influx. Since the unidirectional fluxes of exchange equilibrium fluxes c and d are equal, this permits the following simplification:
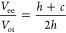
2

Given that at 24 °C, *c*/*h* ≈ 10, the observed ratio of *V*_ee_/*V*_oi_ increases from 5.5 to around 100 when the temperature is reduced to 0 °C for both d-glucose net and exchange flux rates in rat and human erythrocytes.^29,30^ The ratio of inward to outward movement of the empty carrier (*g*/*h*) is estimated to increase from ≈10 at 24 °C to ≈200 at 0 °C.^22,24^ It should be noted that at ≥37 °C, *V*_ee_/*V*_oi_ ≤ 1.^26,32,33^

Agreement between the SMCM predictions and experimental findings diminishes when exchange rates between different pairs of hexose epimers in human erythrocytes are compared, e.g., between d-glucose and mannose or galactose^20,30^ and 3-*O*-methyl d-glucose (3-OMG), 2-deoxy-d-glucose (2DOG) and D-mannose in rat erythrocytes.^29^ As the unidirectional rate of the carrier loaded with mannose or 2DOG is slower than for 3-OMG, the expectation is that mannose homo exchanges should be slower than for 3-OMG. However, this is not observed in practice; the exchange rates are similar.

Another experimental finding indicative of a differing mechanism for the net and exchange flux is that the Arrhenius activation energies of exchange and net flux differ substantially. Comparisons of net d-glucose efflux and exchange flux across human erythrocyte membranes show that the activation energy above 27 °C ≈ 90 kJ mol^–1^ for net flux, while for exchange flux it is 60 kJ mol^–1^. Below 24 °C, the activation energy for *net flux* increases to 150 kJ mol^–1^ and for exchange flux to ≈120 kJ mol^–1^.^32^ Additionally, on raising the temperature from 0 to 20 °C, the *Q*_20_ is 5.38 for net influx and for exchange flux is 10.4.^28,34^ Lowe and Walmsley deduced that the divergence of these parameters at low temperatures is due to the higher activation energy of the outward movement of the unloaded carrier than for the loaded carrier (172 ± 3.1 vs 31 ± 5.1 kJ mol^–1^, Scheme 1, rate h vs rate d). Below 23 °C in rat erythrocytes, the activation energy for the outward movement of the unloaded glucose carrier was 200 ± 25 kJ mol^–1^, and above 23 °C, 101 ± 7.5 kJ mol^–1^.^35^ This large increase in activation energy at low temperatures was ascribed to decreased membrane fluidity rather than to the putative slow return of the empty carrier.

**Scheme 2:**
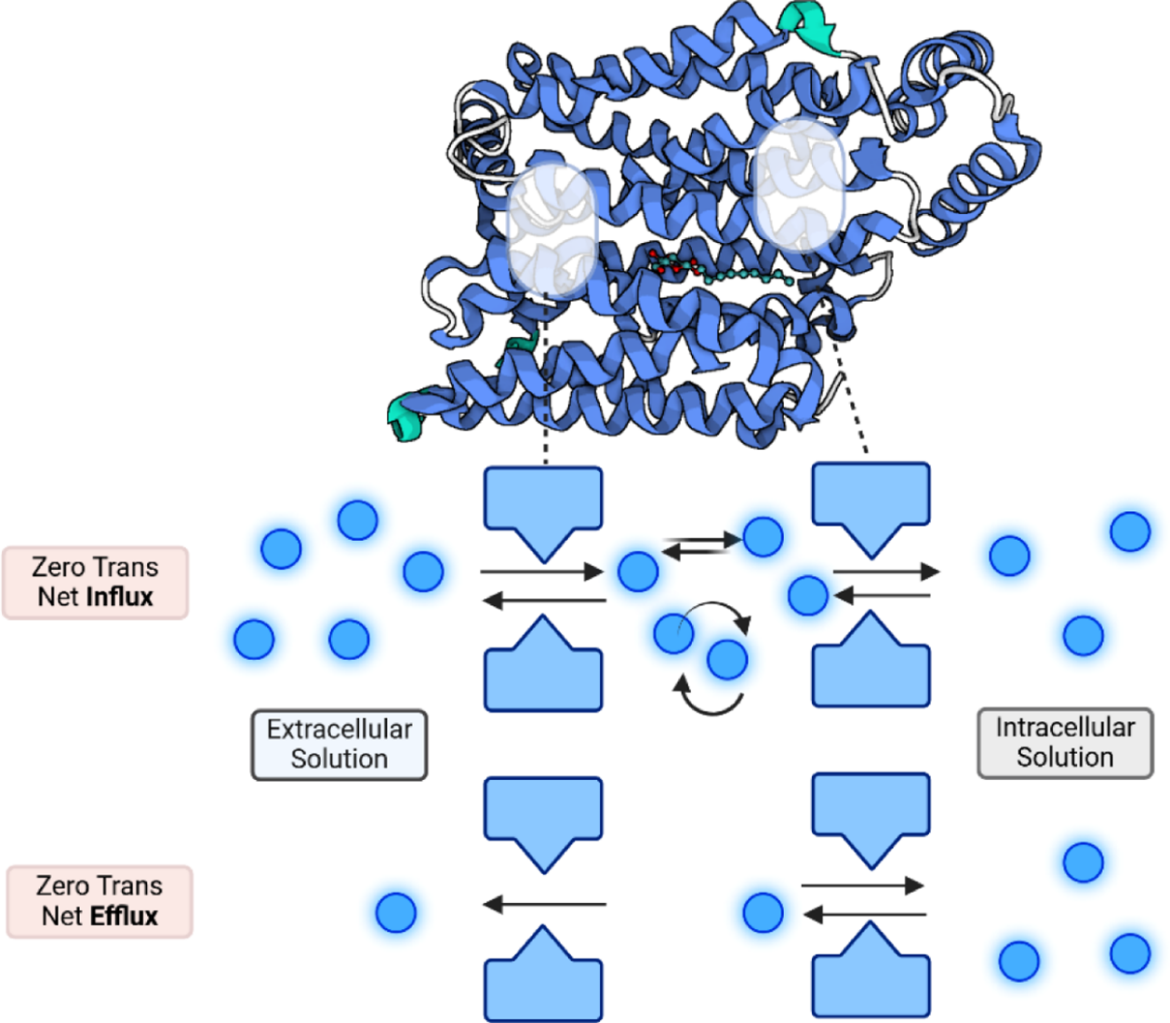
Cartoon Representation Illustrating Zero-t*rans* Net d-Glucose Influx Into Erythrocytes with Saturating d-Glucose in The External Solution And Low Concentration of d-Glucose in the Cytosol. Hypothetically, d-glucose molecules accumulate in the internal vestibule of the protein proximal to the internal bottleneck, where self-exchanges and the ligand accretion further retard net influx. In contrast, in zero*-trans* net efflux, the vestibule is depleted of ligand, so exit via the external bottleneck is unhindered. This scheme was created in BioRender (Domene, C. (2025) https://BioRender.com/d07b866).

Overall, these results indicate that the mechanisms of net and exchange transport differ and that the existing alternating access model predictions are an inadequate description of the exchange transport. Alternative models, based on fixed glucose binding sites at the membrane surfaces with ligand diffusion between the two opposing interfaces (as shown in Scheme 2), were rejected for two main reasons: first, at that time, no compelling evidence supported such a complex view of transport;^16,18^ and second, the absence of carrier mobility would result in the simultaneous occupation of two fixed sites at the membrane surfaces by transportable ligands, thereby inhibiting transport.^14,36^

Superficially, the double-gated variant (DGV)^8,37^ of the alternating access model of glucose transport is equivalent to the SMCM in that the observed ligand-operated gate openings and closures allow d-glucose to pass selectively through GLUT1, while maintaining isolation between the phases on either side of the membrane. However, DGV differs from SMCM in two important ways. The presence of ligand-operated gates at both ends of the central pore implies that the capacity of the transporter for ligand selectivity is not confined solely to the locality of the central binding site as the SMCM implies but, instead, requires a more globally distributed selectivity. Additionally, the intermediate enclosed space spanning the central pore between its external and internal gates permits d-glucose and water diffusion, rather than if the ligand remains bound to the protein surface and its movements are dependent on the protein conformational changes. Thus, in the DGV, d-glucose transit across the membrane between the solution phases is no longer envisaged as a one-dimensional regulated process, where ligands move as protein-determined steps. The introduction of an intermediate stochastic process in the DGV abandons the explicit SMCM assumption that d-glucose transport conforms to a single cyclic process. The DGV becomes more like the independent multisite models, as discussed in^11,29,38,40^ and aligns with the known structural and dynamic features of GLUT1. A remaining necessary constraint is that glucose activities in all connected intracellular compartments will become equal to those in the external solutions at equilibrium.^41^

Several molecular dynamics (MD) simulation studies have shown that d-glucose movements within the central pore along the protein are not confined to the one-dimensional axis perpendicular to the membrane bilayer. MD trajectories have demonstrated that stochastic three-dimensional d-glucose movements and rotations occur within the protein central pore.^42,43^ We have observed prolonged glucose dwelling times extending for several hundred nanoseconds in both the central region around *Z* = 0 Å and the external vestibule at *Z* = 15 Å. These prolonged stays within the central pore’s cavities are examples of glucose entrapment rather than binding, as the ligand residences can move within the constraints of the cavity boundaries or by cooccupants of the space. Similar observations have been reported by others.^43,47^ These prolonged staged residences are completely uncorrelated with any coordinated “breathing” fluctuations of membrane proteins, which typically occur within a periodicity of 3–5 ns.^53^

This staged stochastic progression of glucose between a multiplicity of sites along the central channel, with the additional trait of two-dimensional motion, which permits ligand bypassing of adjacent ligands, means that the DGV is not simply another version of the SMCM, as it is not cyclical. Consequently, the Haldane constraints no longer apply to this transport model, as the trajectory is not a single unbranched process. Additionally, the occurrence of stochastic three-dimensional glucose movements within the central cavities of GLUT1 introduces the possibility of multiple ligand co-occupancy and concurrent, rather than sequential, binary exchanges, as required by the SMCM.^11,44,45^

A single docked d-glucopyranose in the central high-affinity site of GLUT1 increased the probability of the extracellular gate widening sufficiently to permit entry of d-glucose into the central channel in reported simulations.^46^ This simulated condition is assumed to reproduce the effect of saturation achieved by the infinite-*trans* condition. These simulations support the view that allosteric interactions between the central binding site and the external gate could explain the accelerated exchange. Here, we used additional MD protocols to demonstrate the existence of simultaneous β-d-glucose exchanges within GLUT1.

## Material and Methods

### Molecular Dynamics Simulations

Molecular dynamics simulations of GLUT1 with docked β-d-glucose were performed using the crystal structure of the human d-glucose transporter GLUT1 (PDB ID: 4PYP) as a starting point.^37^ The reported bound nonyl β-d-glucopyranoside to the crystal was removed. The initial systems were generated by using the Membrane Builder module of CHARMM-GUI. GLUT1 was inserted in a 1,2-dipalmitoyl-*sn*-glycero-3-phosphocholine (DPPC) patch. In this study, we employed a DPPC lipid bilayer as the model membrane, providing a simplified, homogeneous environment to study the specific dynamics of GLUT1. While the DPPC bilayer does not replicate the full lipid diversity and asymmetry of native cellular membranes, it offers a controlled setting in which to explore the foundational mechanisms of transport. Although we acknowledge that the unique lipid composition of cellular membranes may influence these processes, this approach enables us to isolate the key dynamics of interest. Future studies with more complex lipid mixtures, reflective of the native environment, will be valuable for further contextualizing these findings under physiological conditions.

**Figure 1:**
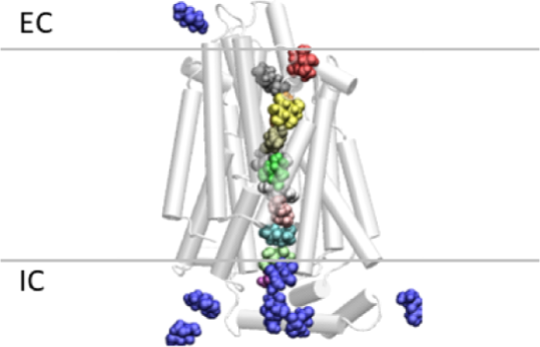
Cartoon representation of GLUT1 showing docked glucose molecules along the pore axis, colored differently, with those initially present in the bathing solution depicted in blue; this is an example of what we termed “saturated-flooded”. Initially, 14 glucose molecules were docked along the pore axis, and other 48 were randomly placed in the solution. The horizontal lines represent the boundaries of the cell membrane at the extra and intracellular sides (EC/IC), respectively.

Previously, we described MD simulations where a protocol denoted as “flooding” was applied^43^ the external solutions are “flooded” with the mobile ligands, which are allowed to partition into the membrane and/or protein sites during the MD trajectory.^47^ An additional protocol has also been adopted here to increase the frequency of intramolecular ligand exchange events and opportunities to observe whether the exchange of positions occurs between d-glucose molecules in adjacent positions because of the scarcity of d-glucose binding events at adjacent positions in the flooded protocol. This alternative protocol is denoted “flooded + saturated” or “flooded + docked”; β-d-glucose molecules are docked at positions extending from the external vestibule to the internal vestibule in the former^48^ or just in the central binding site in the latter. Here, in total, 56 β-D -glucose molecules are present when the “flooded + saturated” protocol is followed, 12 of which initially occupy the “saturated” central pore when the “saturated” protocol is applied. The initial simulation system is shown in Figure 1. In the saturated condition, after becoming fully equilibrated, the d-glucose concentration in the external solution is equivalent to approximately 5–10 mM, depending on the proportion of the total d-glucose ligands remaining within the transporter, whereas in the “flooded + saturated” condition, the equilibrated glucose concentration is between 50 and 60 mM.

The combined system was solvated with an equivalent concentration of KCl 150 mM to produce an electroneutral rectangular simulation box of dimensions 116 × 116 × 120 Å^3^. The CHARMM36 force field^49^ was selected to model the protein and lipids, standard CHARMM parameters were used for ions,^50^ and the TIP3P model was used for water.^51^ Pressure was maintained at 1 atm with a Langevin piston,^52^ using a damping time constant of 50 ps and a period of 200 ps. Temperature was maintained by coupling the system to a Langevin thermostat with a damping coefficient of 1 ps^–1^.^53^ Two temperatures were employed, a higher (50 °C) and lower (35 °C) temperature than the gel-to-liquid crystalline phase transition of DPPC (41 °C), as previously described.^45^ The particle mesh Ewald (PME) algorithm^54^ was used to evaluate electrostatic interactions beyond 12 Å, with a PME grid spacing of 1 Å, and NAMD defaults for spline and κ values. A cutoff at 12 Å was applied to nonbonded forces. Both electrostatics and van der Waals forces were smoothly switched off between the switching distance of 10 Å and the cutoff distance of 12 Å, using the default switching function in NAMD. A Verlet neighbor list with a pair-list distance of 13.5 Å was used to evaluate nonbonded neighboring forces within the pair-list distance. The lengths of covalent bonds involving hydrogen atoms were constrained using the SHAKE algorithm to use a 2 fs time-step.^55^ The multitime step algorithm Verlet-I/r-RESPA was used to integrate the equations of motion.^56^ All the systems were subject to 10,000 steps of energy minimization, followed by an equilibration period consisting of the sequential release of various restraints added to the system: (i) harmonic restraints to heavy atoms of the protein and ions, (ii) repulsive restraints to prevent water from entering the hydrophobic region of the membrane, and (iii) planar restraints to hold the position of the lipid headgroups along the *z*-axis. Subsequently, production runs were executed at the selected temperatures using the NAMD2.13 software.^57^ Simulations were run in triplicates. A summary of the simulations considered in this study is presented in Table 1.

**Table 1:** Details of the MD Simulations Performed in this Study

System Notation	Temperature (°C)	Lipid Phase	Simulation Time [μs] **×** #Replicas^a^^a^	**#**d**-**glucose
Saturated-Flooded Fluid (SF)	50	Fluid	1 × 3	56 (44 + 12)
Saturated-Flooded Gel (SG)	35	Gel	1 × 3	

a Note: Replica 1 in fluid phase was run up to 2 μs.

## Results

**Figure 2:**
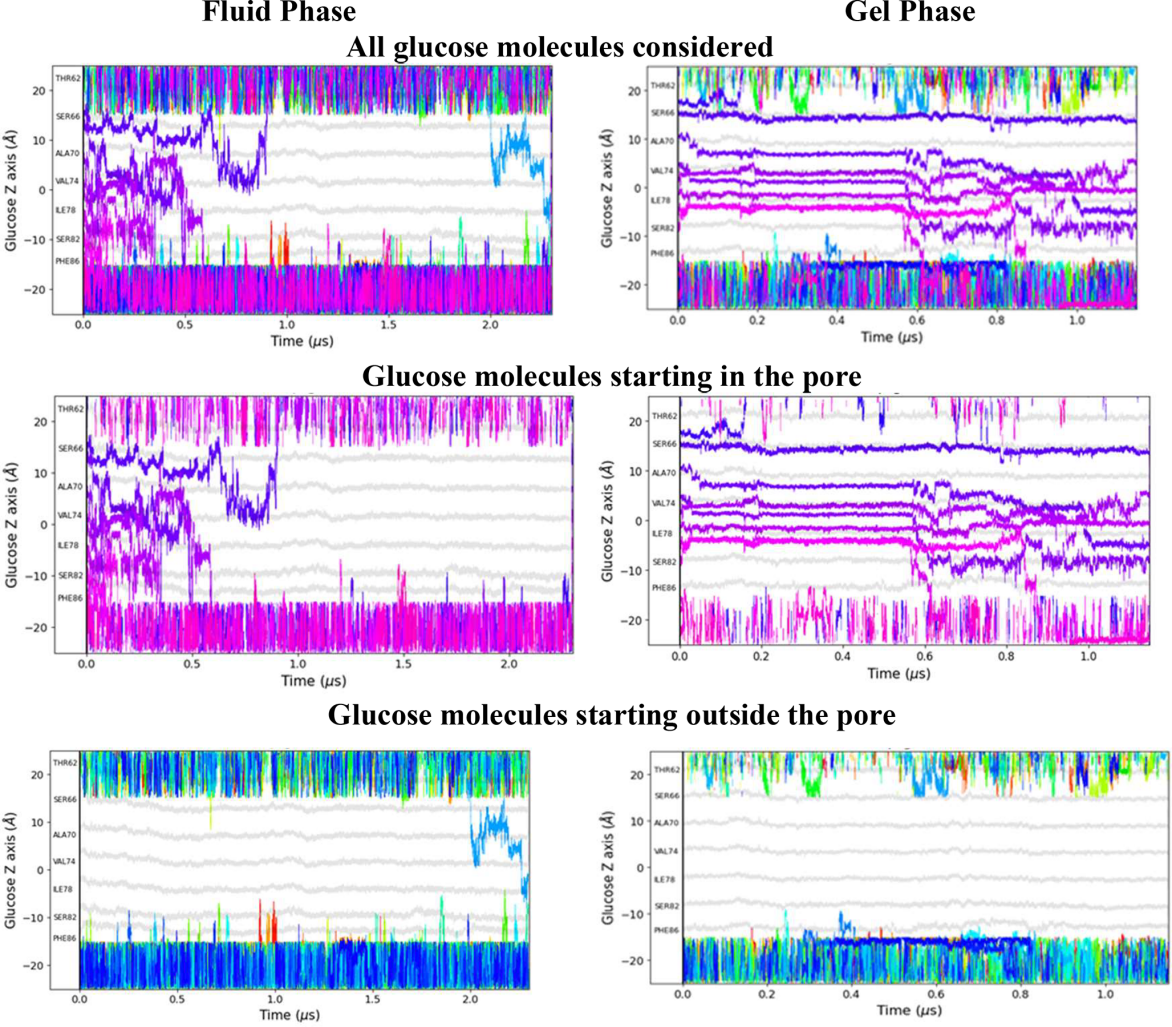
Evolution of the positions of the center of mass of different d-glucose molecules either along the main pore of the protein or outside the protein in the surrounding cytosolic or external media for one of the replicas in either the fluid or gel phase. The origin of the *Z*-axis corresponds to the center of mass of the lipid membrane. The gray lines indicate the positions of the Cα atoms of GLUT1 amino acids lining the central pore. Each color trace corresponds to a different d-glucose molecule. The first row shows plots illustrating glucose molecules both docked and in solution, while the subsequent rows display only those molecules docked inside the protein or those initially in solution at the start of the simulation, presented independently for clarity.

Previously, we demonstrated through in-silico simulations that multiple d-glucose molecules can simultaneously infiltrate into the intramolecular cavities of GLUT1 when embedded in fluid DPPC membranes, but not under gelled conditions.^42,45^ At lower temperatures, in the membrane gel state, fewer d-glucose molecules enter the intramembranous domain.^45^ The current aim of the flooded-saturated protocol in this paper is to fill GLUT1’s intramolecular spaces with d-glucose ligands and to determine whether, after diffusing from their initial positions, the ligands can exchange positions by circumventing or side-stepping each other, either through close apposition ≤3.5 Å or wider separation ≥3.5 Å. The results show that even when the membrane is in the fluid state, d-glucose molecules can remain relatively stationary for prolonged periods ranging from 0.15 to 0.6 μs. When glucose is absent from the external solution, glucose molecules initially docked in the external vestibule of the protein (between *Z* = 5–15 Å, see Figure 2a, where *Z* = 0 Å corresponds to the center of mass of the membrane) have a shorter residence time within the central TM region under fluid-saturated conditions compared with when glucose is initially present in both the external solution and the docked intramolecular positions. This suggests that the exit via the transporter’s external surface is hindered by interactions with the glucose molecules in solution. However, there is no evidence of inhibition of glucose exit across the internal transporter surface in either fluid or gel states. d-glucose mobility across the internal surface is greatly increased by the presence of saturating d-glucose ligands in the internal solution (see below). The ligands docked in the inner intramembranous core of the transporter, from *Z*= −20 to +15 Å, escape to the internal solution within 0.9 μs. Under both fluid and gel conditions, saturated-flooded ligands have an entry rate into the extramembranous zone that is 10 times greater than the flooded condition alone, as presented in Figure 2 of ref.^45^. In the saturated-flooded gel, d-glucose penetrates the transporter more often and to a greater depth, *Z* = +10 Å (Figure 2b). This indicates that the combined effect of multiple glucose molecules within the transporter and the external solution increases d-glucose mobility, particularly in the extramembranous zones. This results in more frequent collisions between adjacent ligands within the central channel under both the fluid and gel states in the “flooded + saturated” protocol compared with either the saturated or flooded alone protocols.^44^

In saturated fluid conditions, all the docked d-glucose molecules diffuse from their initial position toward the cytosolic or the external solution within 0.5–1.0 μs, as illustrated in Figure 2a. In contrast, except for those glucose molecules initially docked in the internal extramembranous linker region between TMs 6 and 7, intramolecular glucose diffusion in saturated gel conditions is much slower (Figure 2b). Centrally positioned intramembrane glucose movements are negligible during the initial 1.5 μs of the trajectory.

In the fluid membrane state, during the initial 0.5 μs, two or more ligands are simultaneously present at the same *Z*-position within the pore. In the gel state, despite the slower diffusion rates of d-glucose, d-glucose exchanges are still observed. It is noteworthy that even under gel conditions, glucose mobility is greatly increased by flooding the external solution with glucose.

During the first 100 ns, glucose molecules diverge from their starting positions and make close contact with each other. When glucose molecules are present at a high density within the central protein regions, they collide and can exchange positions. Exchange occurrences between glucose ligands are observed in the central zone between *Z* = −15 and +12 in the period between 0.1–0.9 μs. Many instances of simultaneous ligand exchanges are observed in both gel and fluid membrane states in the flooded -saturated condition when multiple ligands are present (Figure S1).

The reason for performing atomistic molecular dynamics simulations with the “saturated + flooded” protocol is to investigate whether and how glucose molecules exchange positions within GLUT1 and provide an answer to the question of whether the glucose transporter behaves like a ping-pong enzyme, where the forward and backward movements of the ligand successively traverse aided by conformational changes within the transporter, or as predicted by the multisite model, exchange occurs by simultaneous bimolecular ligand interactions in the protein void volumes. Exchanges of glucose molecules along the pore axis were analyzed throughout the trajectory. An exchange is defined by an initial configuration in which two glucose molecules, *A* and *B*, are oriented along a rotational axis. Each glucose molecule is compared to every other molecule, and each frame is compared to all of the other frames. If glucose *B* shifts to a positive *Z*-axis position in a subsequent frame, with a *Z*-coordinate greater than 0.4 (normalized), an exchange is counted—regardless of hydrogen bonding interactions. To avoid overlap, only the smallest nonoverlapping timeframes for each transition are included. The time point for each exchange is recorded as the midpoint between the two frames, and the *Z*-axis position is calculated as the mean *Z*-coordinate of the centers of mass (CoM) of both glucose molecules across the frames (i.e., the mean of four *Z*-coordinates).

**Figure 3:**
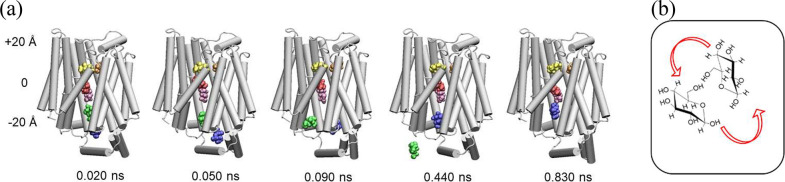
Glucose exchanges within the central channel by rolling H-bonding interactions. (a) Several glucose molecules exit the central pore through gaps formed by the highly mobile intracellular linker between transmembrane segments (TMs) 6 and 7, moving into the cytosolic solution. A close exchange is shown in the internal vestibule, where a green glucose molecule bypasses a blue ligand lodged near TM1 and exits into the cytosolic solution. The blue glucose then advances within the channel, moving from *Z* < 0 Å to form a hydrogen bond with the violet glucose at *Z* = −3 Å. (b) Diagram illustrating d-glucose ligands rotating around each other.

Adjacent glucose molecules can slide over one another through frequent hydrogen bonding interactions with adjacent residues (Figure 3). A typical rolling exchange event is demonstrated within the wide internal vestibule. In a close exchange shown in the internal vestibule, a green glucose molecule bypasses a blue ligand lodged near TM1 and exits into the cytosolic solution. The blue glucose then advances within the channel, moving from *Z* < 0 Å to form a hydrogen bond with the violet glucose at *Z* = −3 Å. This type of exchange requires sufficient space within the protein for two ligands to be juxtaposed both sideways and lengthwise along the *Z*-axis. These exchanges only occur in regions with adequate space, specifically in the upper and lower vestibules and in the interstitial spaces within the extramembranous linker regions.

**Figure 4:**
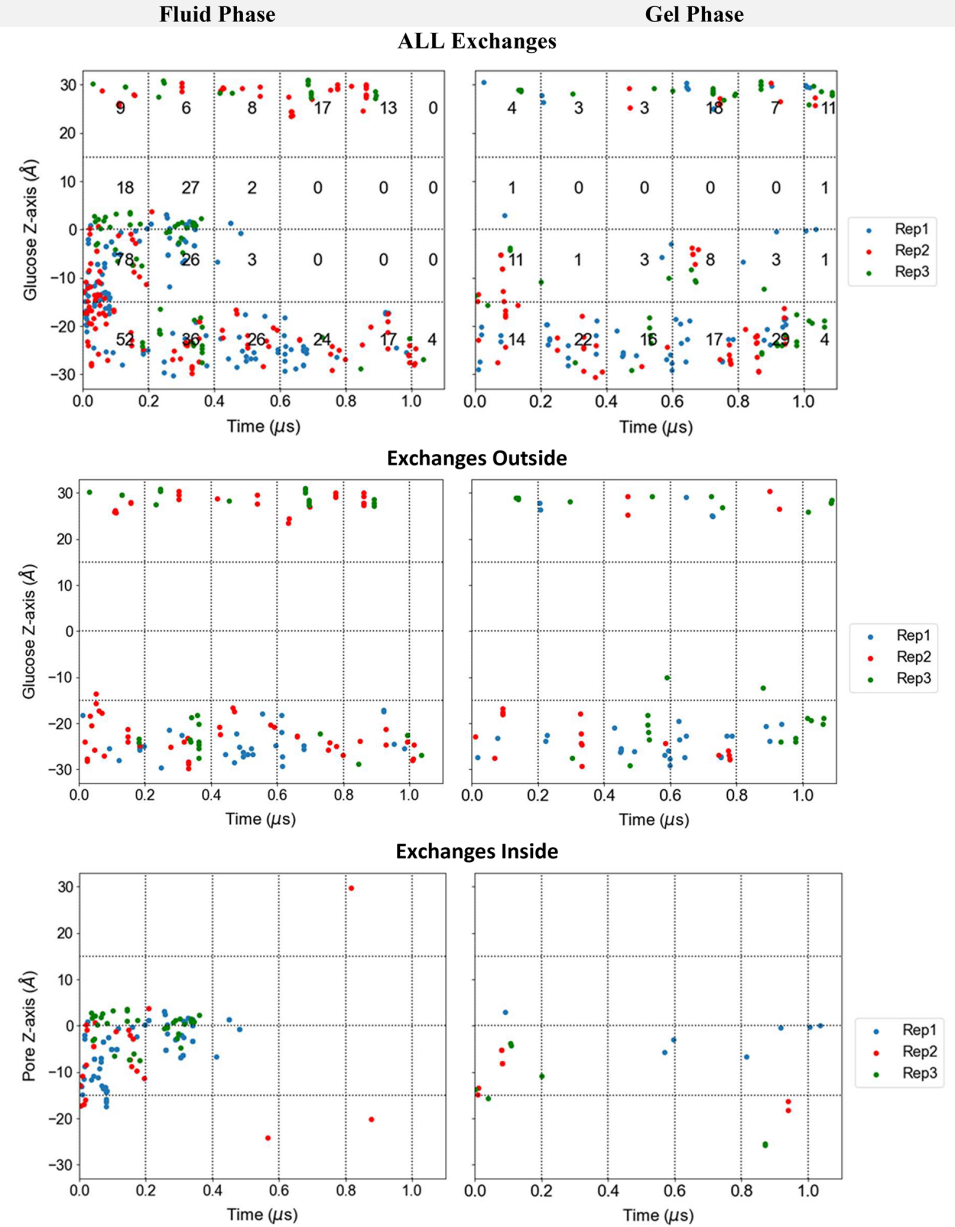
Exchanges of glucose molecules along the pore axis were analyzed throughout the trajectory. An exchange is defined by an initial configuration where two glucose molecules, A and B, are oriented along a rotational axis. Each glucose molecule is compared to every other molecule, and each frame is compared to all other frames. If glucose B shifts to a positive *Z*-axis position in a subsequent frame, with a *Z*-coordinate greater than 0.4 Å (normalized), an exchange is counted—regardless of hydrogen bonding interactions. To avoid overlap, only the smallest nonoverlapping timeframes for each transition are included. The time point for each exchange is recorded as the midpoint between the two frames, and the *Z*-axis position is calculated as the mean *Z*-coordinate of the centers of mass (CoM) of both glucose molecules across the frames (i.e., the mean of four *Z*-coordinates).

Figure 4 illustrates the evolution of exchanges in each replica over the simulation time for both the fluid and gel phases. There are numerous exchanges occurring in the fluid system (Figure 4, left panels) before all docked glucose molecules exit the pore, as they move more freely within the pore compared with the gel phase system, where they exit prior to 0.4 μs.

**Figure 5:**
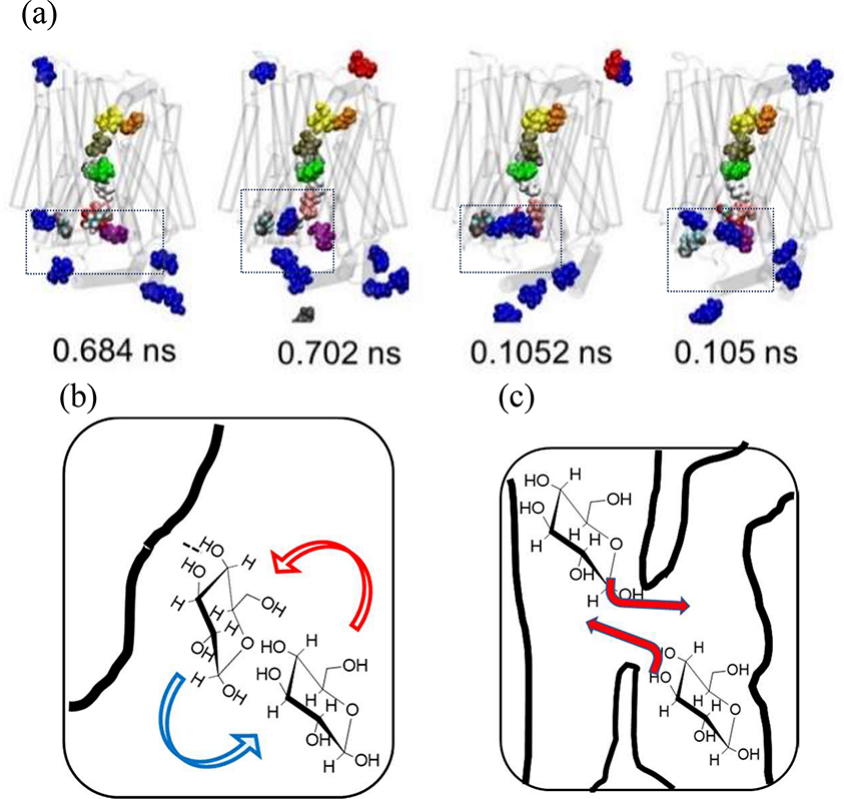
Glucose surface or bypass exchanges: glucose molecules in solution either bind to surface-bound glucose molecules, displacing them in situ, or bypass without making contact. Glucose molecules initially in solution are represented in blue, while docked molecules are shown in a contrasting color. (a) The framed region highlights glucose ligands undergoing transient bypassing. The cyan-colored, surface-bound glucose is shown being displaced from its binding site. Illustrations depict (b) surface exchange, where a bound glucose molecule is replaced by another in the adjacent solution, and (c) bypassing, where two glucose ligands travel independently without making.

Another commonly observed type of exchange is where surface-bound glucoses exchange simultaneously with incoming ligands from the surrounding solution, or where the ligands bypass one another with “wider” separations without touching, via parallel channels or tunnels within the protein (Figure 5). These types of exchange indicate that when multiple ligands are present within the same transporter pathways, there is no absolute hindrance preventing neighboring ligands from intermingling, as would occur if these movements were constrained to single-file, one-dimensional flows.^36^

## Discussion

The primary aims of this study are to understand and illustrate, at an atomistic level, how d-glucose molecules exchange within GLUT1. The data presented demonstrate that simultaneous exchanges of d-glucose positions occur between adjacent molecules, both within the intramembranous regions of GLUT1 and at its solution interfaces. These simulations show that d-glucose is free to move and rotate within the intramolecular vestibules. Contiguous d-glucose molecules slide across the surface of their exchanging partner. Small deviations in d-glucose positions occur in both the vertical and horizontal planes, allowing for their transposition along the pore axis. These three-dimensional movements involve multiple hydrogen bond interchanges between the overlapping d-glucose molecules and exert mutual drag forces that can draw the protein-bound molecule from its binding site.

Intramolecular glucose exchange events, where both ligands are encased by the protein, occur more slowly, with durations ranging from 0.002 to >0.02 μs, compared to the exchanges at the solution-protein interfaces, where ligand displacement of surface-bound d-glucose by more mobile solution-derived d-glucose generally occurs at a faster rate (within 0.001–0.003 μs).

The significance of these findings is that the exchange events observed in these MD simulations do not align with the sequential process envisaged by the SMCM, where ligands are impelled by protein conformational changes at the central high-affinity binding site. Instead, simultaneous exchanges occur at multiple loci within both the intra- and extramembranous zones. Intramolecular exchanges also take place when widely separated glucose ligands bypass each other along the *Z* plane, as if negotiating a two-lane highway.

As originally conceived, SMCM contains a single mobile binding site for d-glucose without any provision for multiple binding sites or ligands, so with this model, glucose exchange can only be accomplished by sequential, unidirectional cross-membrane strides.^11^ This model prediction has been confounded by binding studies with purified GLUT1 showing that two mol of nonmetabolized d-glucose derivative 3-*O*-methyl-glucoside (3-OMG) is bound per mole of GLUT1 with ATP + Mg^2+^ (4 mM) present. Without ATP, only one mole of 3-OMG per mole of GLUT1 is bound.^58,59^ ATP interactions confine the additional hexose ligand within the structured endofacial loop between TMs 6–7 and the C-terminal domain, in both GLUT1^3,60^ and GLUT4.^61^ As GLUTs have been shown experimentally and *in silico* to accommodate multiple d-glucose ligands,^43,46,59,62^ it follows that transport mechanisms based on the hypothesis that d-glucose transport requires a single mobile central high-affinity binding site are insufficient. The presence of both multiple ligands and low-affinity binding sites within the transport pathway negates the hypothesis that transport conforms to a single unbranched cyclic pathway, as required by the SMCM.^38^

Additionally, the concept that transmembrane flows of radioisotope-labeled ligands represent “unidirectional” or transmembrane fluxes misrepresents how glucose molecules move through GLUT1. This perception of unidirectional transmembrane glucose flow originates from the early view that membrane transporters consist of inside and outside “adsorption” surfaces^14,18^ without any intervening structures. Structural studies of GLUTs have shown that this early concept is incorrect.^6−9^ Kinetics based on estimates of initial rates of radioactive glucose tracer can only provide a good estimate of the “unidirectional” glucose rate if ligand traversals through the transporter are unimpeded by ligand aggregates or temporary bottlenecks within the central channel. The concept that unidirectional flux is a direct measure of ligand transmembrane permeability implicitly assumes that tracer back fluxes solely arise from external sources, e.g., from tracer returning from contralateral adsorbed or “unstirred” layers, or from the *trans* solution itself.

The MD depictions of glucose transport via GLUT1 show that individual ligand trajectories do not perform as unidirectional flows, as demonstrated here. Instead, the trajectories consist of three-dimensional, multistep, stochastic chain processes, where multiple ligands can interact simultaneously.

Molecular dynamics simulations have the unique capacity to demonstrate intramolecular glucose exchanges occurring between molecules of the same type as well as differentially labeled types. As in MD, all the glucose molecules are individually defined, this provides the facility to tally all exchanges occurring, irrespective of whether they are otherwise labeled or not. The capacity to accommodate multiple molecules and 3D movements with accretion formation proximal to bottlenecks generates alterations in glucose’s trajectory that lead either to apparent acceleration of exchange flow when occurring between different isotopes, or retardations when the exchanges occur between the same isotope type and are latent.

Geminate exchanges formed within intramolecular ligand accretions provide a simple explanation for the relative temperature insensitivity of intramolecular exchange glucose rates in GLUT1 in comparison with the high temperature sensitivity of net flux. The exchange frequencies depend on the numbers and durations of intramolecular ligand accretions. These are increased by prolonged closure of the bottlenecks that result from the membrane gel formation at low temperatures. The bottlenecks obstruct net glucose flows between the external solution and the central channel.^47^ However, as intramolecular exchanges still occur in the channel cavities that remain open during bottleneck closures in the gel state, this explains why the observed exchange rates are less retarded at low temperatures than is net flux.^26,43,47^

Polarized intramolecular ligand accumulation at the internal bottleneck during net d-glucose influx accounts for the observed differences in flux parameters between the low *V*_max_ and *K*_m_ for net entry and high *K*_m_ and *V*_max_ for net exit,^3,38,63^ where ligand accretions accumulating proximal to the internal bottleneck slow net ligand exit to the internal solution. Conversely, zero-*trans* net exit from an intracellular solution loaded with saturating glucose concentrations into a trans solution with zero glucose present leads to ligand depletion within the external vestibule (Scheme 2). The absence of any hindrance from ligand accretions within the external vestibule will raise the apparent *K*_m_ and *V*_max_ of zero-*trans* net exit.

Mutations that block transport across the external surface of GLUT1, e.g., T295M, are a rare and scientifically interesting cause of GLUT1 deficiency syndrome. Structural and modeling studies have shown that this is a consequence of the hydrophobic methionine substitution for hydrophilic threonine, which blocks one of the two available portals of the external vestibule, causing retardation of exit flow.^39^ This mutation results in glucose buildup in the external vestibule and slows both d-glucose net uptake and exit from the external vestibule to the external solution,^39,64,65^ providing a straightforward explanation for the large reduction at low temperatures observed in the *V*_max_ and K_m_ of zero-*trans* net exit of d-glucose by the GLUT1 mutation, T295M.^66,67^

### Accelerated Exchange

**Scheme 3:**
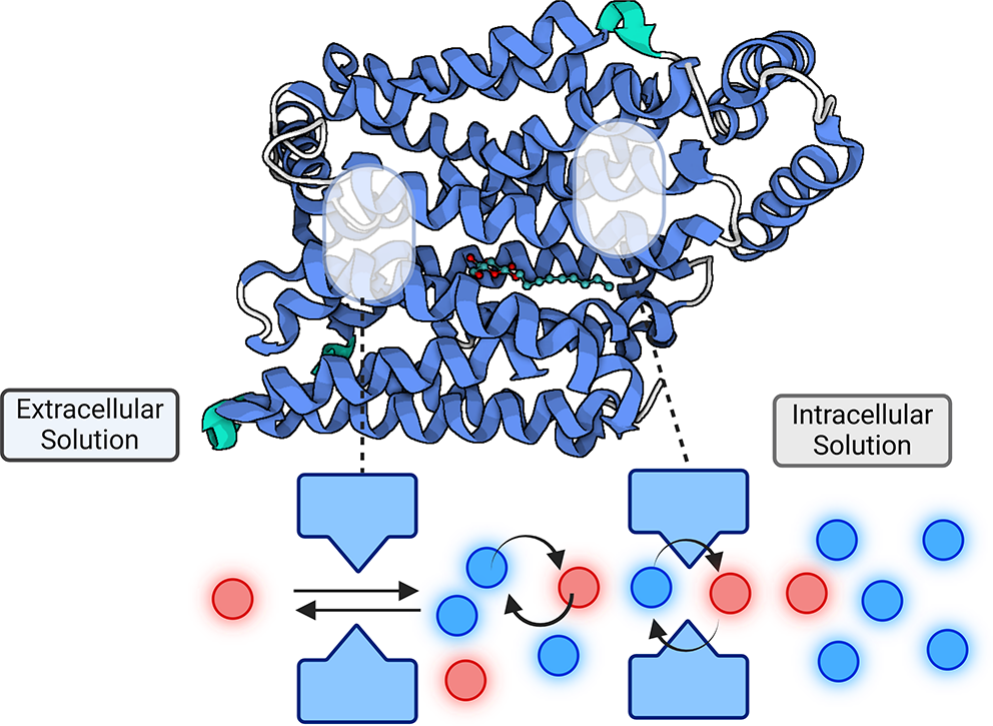
Cartoon Illustrating Infinite *Trans* Exchanged-glucose Influx via Glut1 with a Saturating Concentration of Unlabelled d-Glucose in the Cytosolic Solution (Blue Circles) and Low Concentrations of Labelled d-Glucose in the External Solution (Red Circles) Unlabeled d-glucose molecules (blue circles) accumulate in the internal vestibule proximal to the internal bottleneck, where exchanges between labeled and unlabeled ligands will appear to generate accelerated d-glucose influx as these substitute for latent self-exchanges. The difference between net and exchange flux can be viewed as a function of the frequency at which exchanges between labeled d-glucose substitute for unlabeled d-glucose self-exchanges. While individual exchanges are indeed simultaneous within each event, exchanges in different parts of the transport process occur independently and are not sequential. This scheme was created in BioRender (Domene, C. (2025) https://BioRender.com/d07b866).

Intramolecular accretions with geminate exchanges also explain why the influx of labeled glucose into human erythrocytes is increased by preloading the cells with unlabeled d-glucose and the infinite *trans* exchangephenomena observed by Lacko et al.^26,34^ Geminate exchanges between labeled and unlabeled d-glucose ligands substitute detectable exchanges for latent label–label exchanges, or unlabeled with unlabeled concatenated exchanges, as illustrated in Scheme 3.

In the infinite *trans* influx condition at low temperatures, prolonged closure of the internal bottlenecks promotes the buildup of unlabeled ligand in the intermediate intramolecular spaces enclosed by the bottlenecks. This increases the probability of detectable ligand exchanges within the intramolecular vestibule when cells are exposed to tracer amounts of isotope entering the vestibule from the external solution, thereby resulting in accelerated exchange influx. Raising the temperature to 37 °C or above will increase the open probability of the bottlenecks^43,47^ and hence reduce intramolecular ligand accretions, thereby reducing accelerated exchange flux relative to net influx.

A similar explanation also accounts for the absence of accelerated exchange in GLUT4,^4,5,27,68^ which may result from the relative sparsity of multiple ligand accretions within the more glucose-permeable central pore of GLUT4 than that of GLUT1. GLUT4 has a *V*_max_ for glucose that is 4–5 times larger than that of GLUT1. This higher *V*_max_ of GLUT4 is seen as an intrinsic advantage for insulin-dependent import of d-glucose into muscle but will prevent intramolecular buildup of intramolecular ligand accretions and hence, prevent any observable acceleration of exchange flux. A recent nuclear magnetic resonance (NMR) study investigated ^19^F-labeled glucose derivatives substituted at the 2- and 3-positions of deoxy-d-glucose to measure glucose anomeric exchange transport at 37 °C. The study reported significantly lower rate constants for the α-anomer of 3-deoxy-3,3-difluoro-d-glucose compared with the β-anomer. However, the method is not currently amenable to lower temperature studies, which could provide valuable direct corroboration of the findings from the current *in silico* analysis.

## Conclusions

The work presented here is a prime example of how MD simulations provide a versatile and comprehensive approach for studying detailed molecular mechanisms, in this case, specifically of glucose transport in the GLUT1 transporter, offering valuable insights into its function and dynamics at the atomistic level. Molecular dynamics simulations of GLUT1 saturated with d-glucose have displayed simultaneous d-glucose exchanges both within the intra- and extramembranous domains of the transporter. Several phenomena can now be readily explained to occur due to the buildup of multiple ligand accretions within the transport pathway of GLUTs on the basis that geminate intramolecular d-glucose exchanges occur within these accretions. The explanation of the increasing ratio of the maximal rates d-glucose exchange relative to net influx,^34^ from close to unity at 37 °C to more than 100 when the temperature is reduced to 4 °C, can result from increased accretion of d-glucose molecules behind the bottlenecks at the external and internal boundaries of the central pore at low temperature, while d-glucose mobility within intramolecular cavities remains relatively unchanged. The absence of accelerated exchange at 37 °C can result from the higher rates at which the d-glucose molecules escape from the central pore: this prevents the buildup of d-glucose accretions within the central pore and accounts for the absence of accelerated exchange. This study may explain the absence of accelerated exchange in GLUT4 as it has much higher glucose turnover rates that would prevent intramolecular glucose aggregation. The absence of accelerated exchange in GLUT4^4^,^5,27,68^ may result from the relative sparsity of accretions of multiple ligands within the more permeable central pore of GLUT4, which has a *V*_max_ 4–5 times larger than that of GLUT1. The higher *V*_max_ of GLUT4 is seen as an intrinsic advantage for the net import of d-glucose into muscle. Future studies leveraging MD simulations are likely to further elucidate the impact of temperature and ligand accumulation on transport dynamics, potentially revealing new insights into the differential regulation and efficiency of GLUT transporters in various physiological and pathological conditions.^69^

## Supporting Information Available

Evolution of the positions of the center of mass of different d-glucose molecules either along the main pore of the protein or outside the protein in the surrounding cytosolic or external media in either the fluid or gel phase for Replicas 2 and 3; evolution of the positions of the center of mass of different d-glucose molecules either along the main pore of the protein or outside the protein in the surrounding cytosolic or external media for each replicas in either the fluid or gel phase considering only those glucose molecules which maintain the interaction for ≥40 ns; RMSFs of the saturated and flooded trajectory and flooded alone trajectories under fluid and gel conditions; hydrogen bond analysis: glucose–glucose vs glucose-protein; average hydrogen bond analysis: glucose–glucose vs glucose-water (PDF)

## Abbreviations

MD: molecular dynamics
SLC2A class 1: facilitated glucose transporter member 1
TM: transmembrane
SMCM: simple mobile carrier model
DPPC: 1,2-dipalmitoylphosphatidylcholine
RMSF: root-mean-square fluctuations
