# Supplementary material for: Insight into the Mechanism of d-Glucose Accelerated Exchange in GLUT1 from Molecular Dynamics Simulations
Source: Biochemistry. 2025 Jan 28;64(4):928–39. doi: 10.1021/acs.biochem.4c00502 (PMC11840925; doi:10.1021/acs.biochem.4c00502)
Supplement: Supplementary file 1 — bi4c00502_si_001.pdf [file bi4c00502_si_001.pdf]

Supplementary Information

Insight into the mechanism of D-glucose accelerated exchange in GLUT1 from molecular dynamics simulations.

Carmen Domene[1*], Brian Wiley[1], *Saul Gonzalez-Resines*[1], *Richard J. Naftalin*[2*]

[1]Department of Chemistry, University of Bath, Claverton Down, Bath, BA2 7AY, United Kingdom

[2]*BHF Centre of Research Excellence, School of Medicine and Life Sciences, King's College London, London* SE1 9NH, United Kingdom

Corresponding Authors: Carmen Domene mcdn20@bath.ac.uk & Richard Naftalin richard.naftalin@kcl.ac.uk

Figure S1. Evolution of the positions of the centre of mass of different D-glucose molecules either along the main pore of the protein or outside the protein in the surrounding cytosolic or external media for in either the fluid or gel phase for Replicas 2 and 3. The origin of the *Z*-axis corresponds to the centre of mass of the lipid membrane. The grey lines indicate the positions of the Cα atoms of GLUT1 amino acids lining the central pore. Each color trace represents a different D-glucose molecule. The first two plots show glucose molecules both docked and in solution. Subsequent plots display the evolution of glucose molecule positions along the z-axis, distinguishing between molecules docked within the protein and those initially in solution at the start of the simulation for clarity.

<table>
<tr><th colspan="2">REPLICA 2</th></tr>
<tr><td>Fluid Phase</td><td>Gel Phase</td></tr>
<tr><td colspan="2">All glucose molecules considered</td></tr>
<tr><td>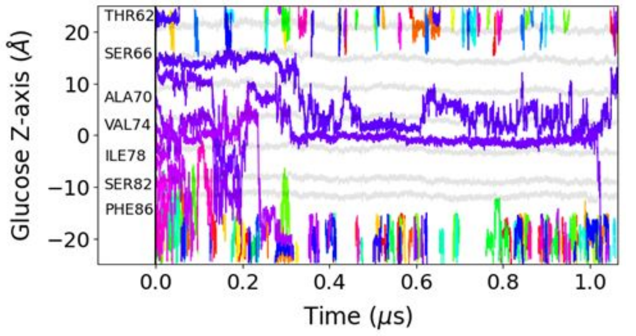
</td><td>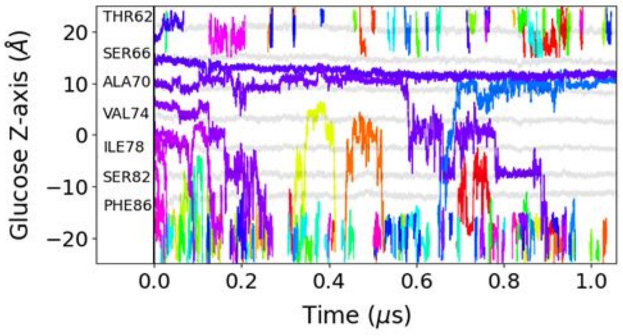
</td></tr>
<tr><td colspan="2">Glucose molecules starting in the pore</td></tr>
<tr><td>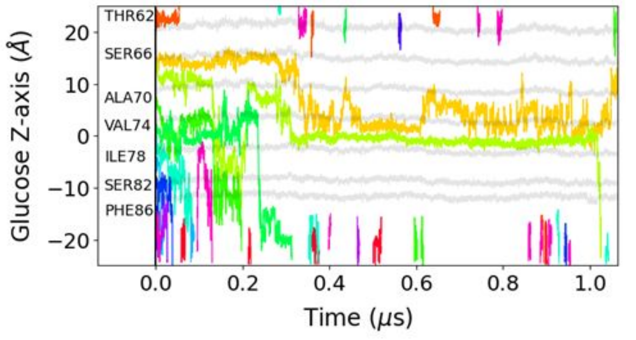
</td><td>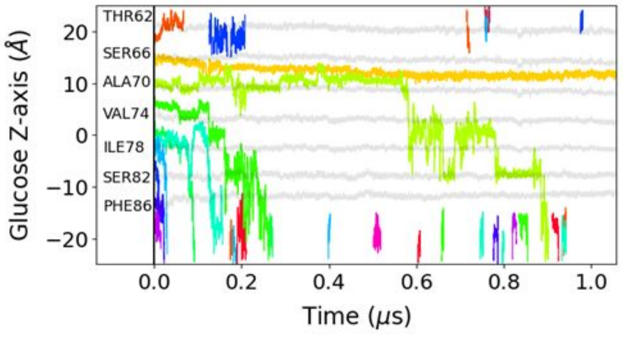
</td></tr>
<tr><td colspan="2">Glucose molecules starting outside the pore</td></tr>
</table>

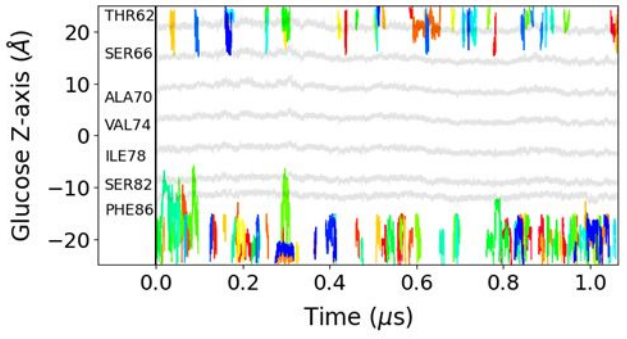

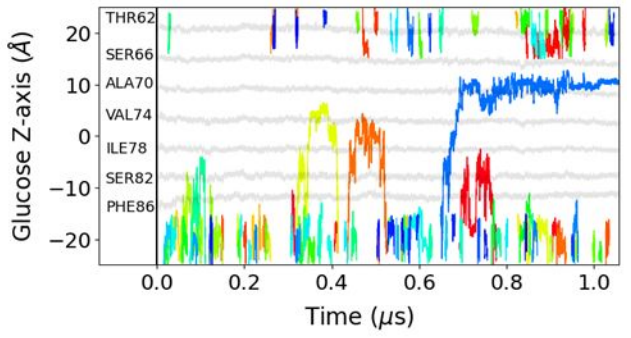

REPLICA 3

Fluid Phase	Gel Phase

All glucose molecules considered

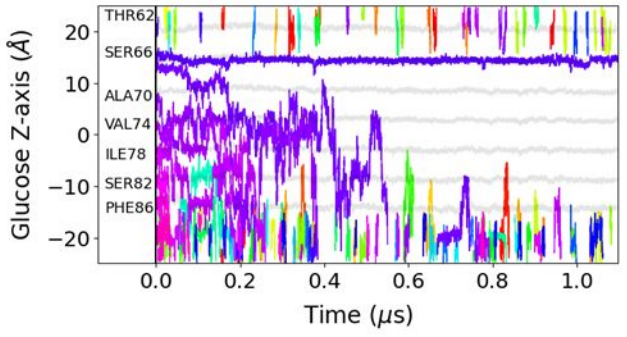

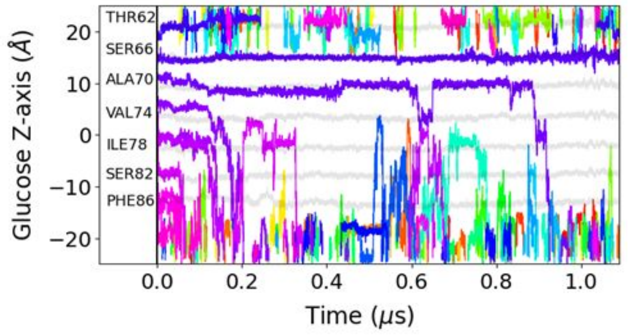

Glucose molecules starting in the pore

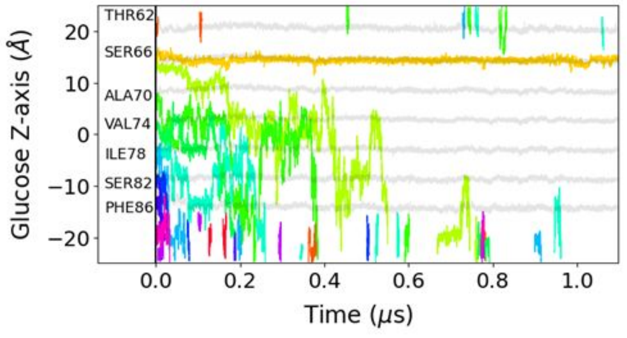

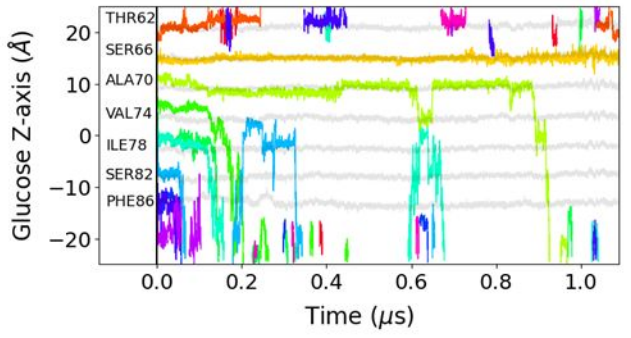

Glucose molecules starting outside the pore

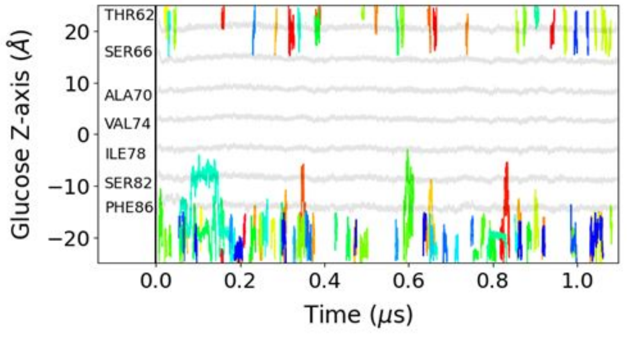

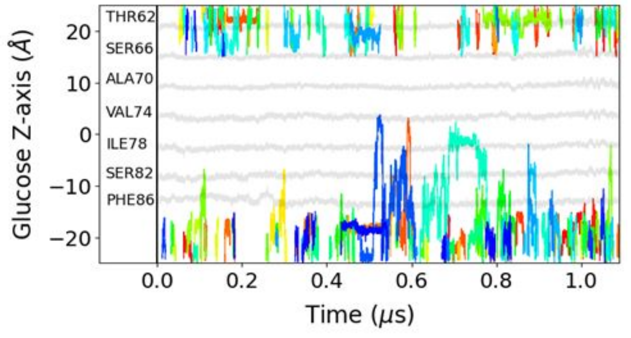

Figure S2. Evolution of the positions of the centre of mass of different D-glucose molecules, either along the main pore of the protein or outside the protein in the surrounding cytosolic or

external media, in both fluid and gel phases. The origin of the Z-axis corresponds to the centre of mass of the lipid membrane, with grey lines indicating the positions of the Cα atoms of GLUT1 residues lining the central pore. Each colour trace represents a different D-glucose molecule that remains in position for 40 ns or longer. The first two plots show glucose molecules both docked and in solution, while subsequent plots illustrate the evolution of glucose molecule positions along the Z-axis, distinguishing between molecules docked within the protein and those initially in solution at the start of the simulation for clarity.

<table>
<tr><th colspan="2">REPLICA 1</th></tr>
<tr><td>Fluid Phase</td><td>Gel Phase</td></tr>
<tr><td colspan="2">All glucose molecules considered</td></tr>
<tr><td>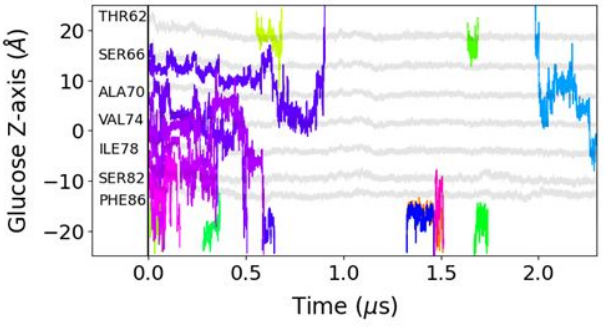
</td><td>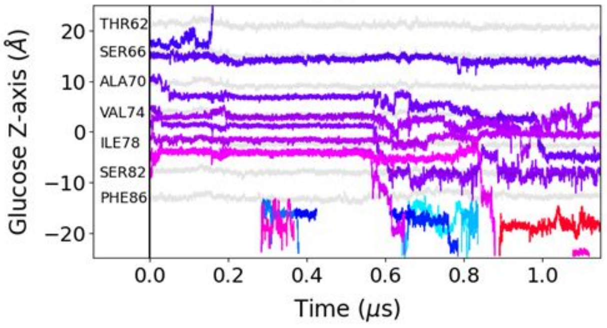
</td></tr>
<tr><td colspan="2">Glucose molecules starting in the pore</td></tr>
<tr><td>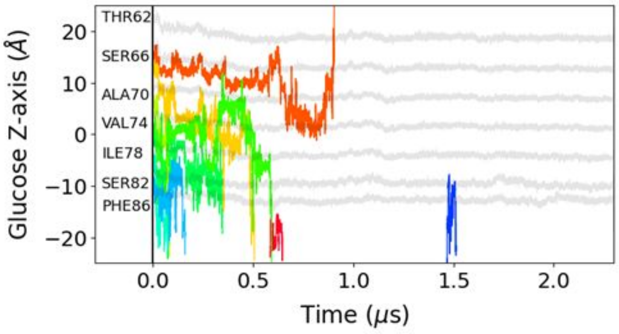
</td><td>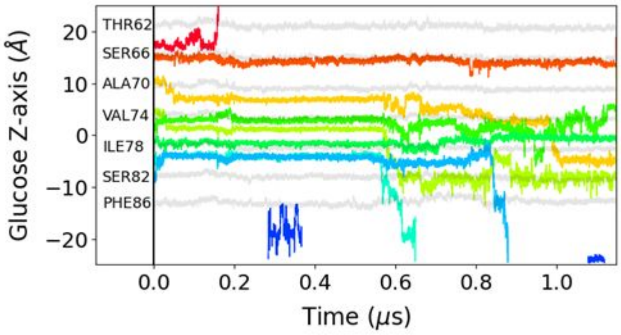
</td></tr>
<tr><td colspan="2">Glucose molecules starting outside the pore</td></tr>
<tr><td>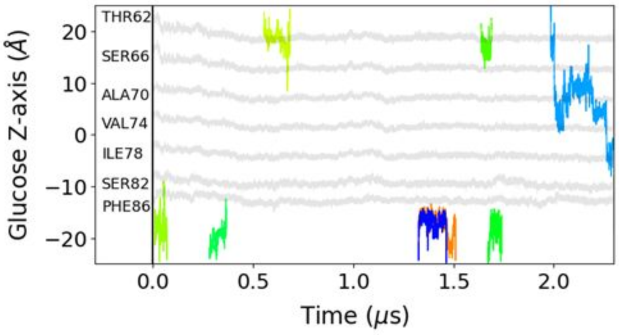
</td><td>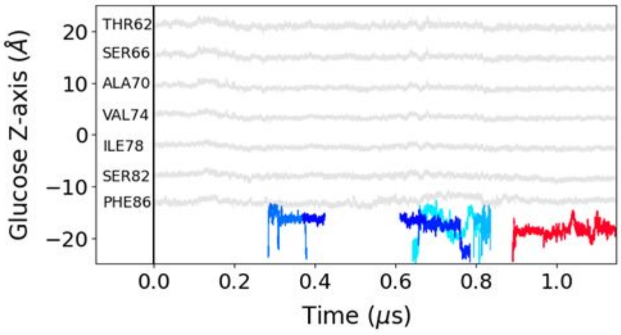
</td></tr>
</table>

<table>
<tr><th colspan="2">REPLICA 2</th></tr>
<tr><td>Fluid Phase</td><td>Gel Phase</td></tr>
<tr><td colspan="2">All glucose molecules considered</td></tr>
</table>

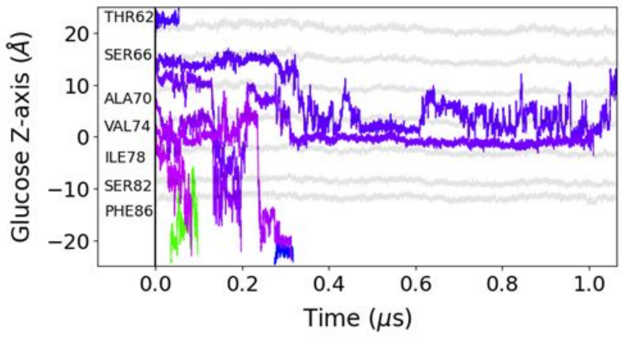

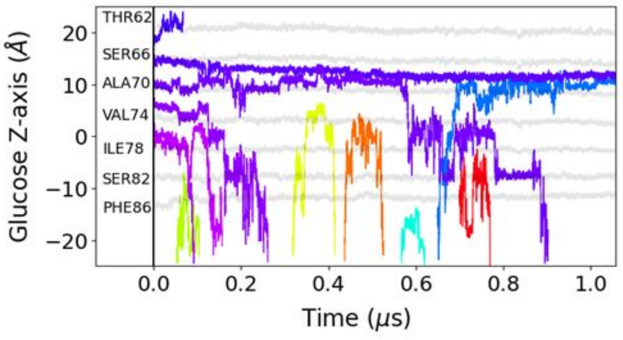

Glucose molecules starting in the pore

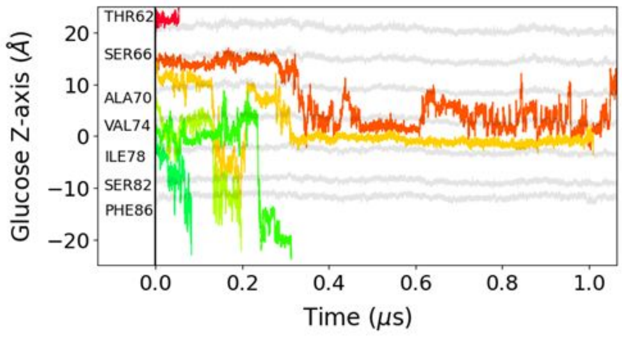

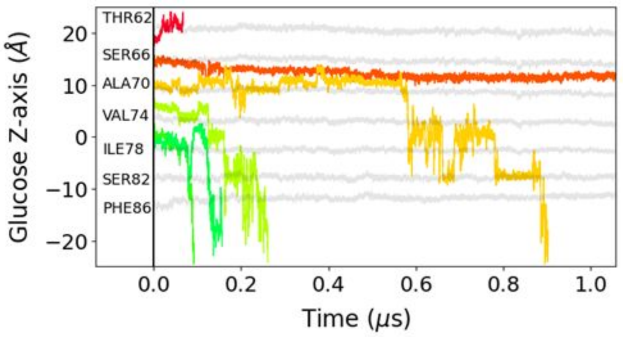

Glucose molecules starting outside the pore

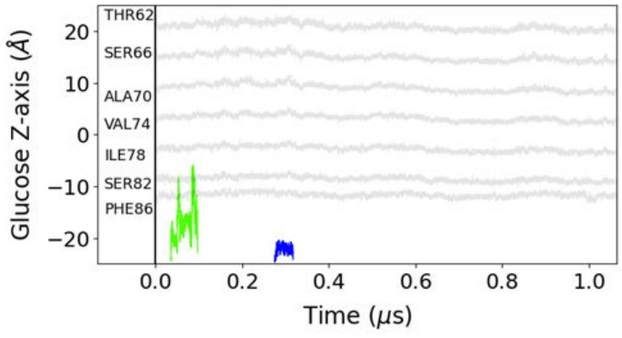

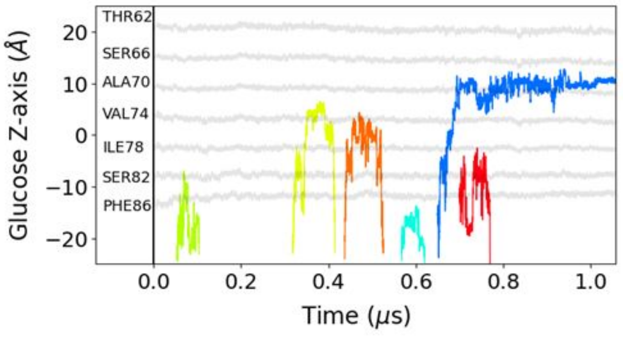

REPLICA 3

Fluid Phase	Gel Phase

All glucose molecules considered

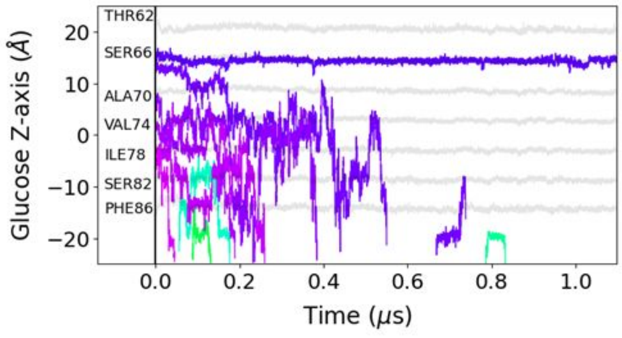

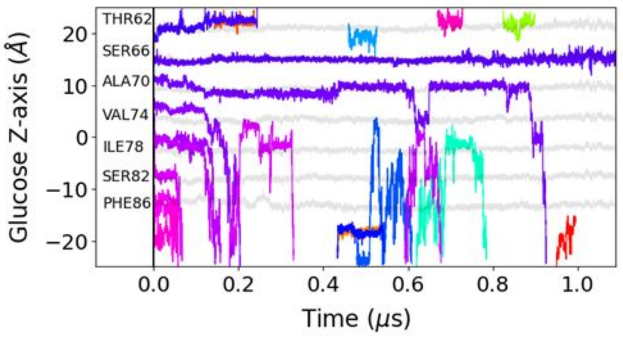

Glucose molecules starting in the pore

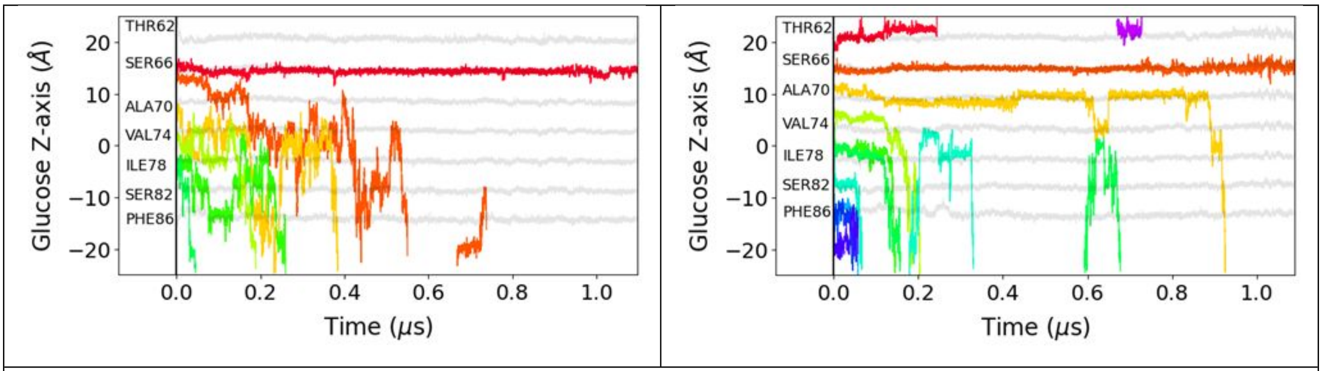

Glucose molecules starting outside the pore

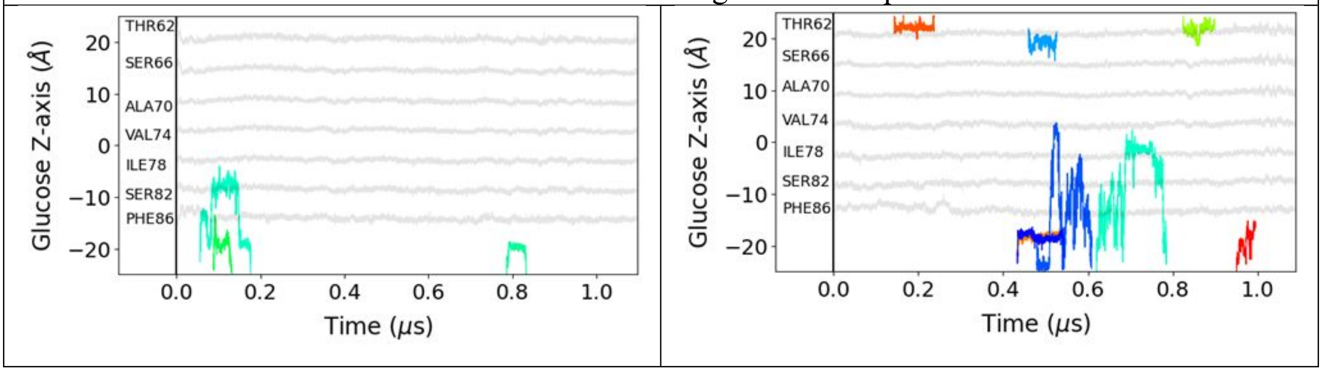

Figure S3. RMSFs of the saturated and flooded trajectory (red) and flooded alone trajectory(blue) under **(A)** fluid and **(B)** gel conditions. The s.e.m. of averaged fluid saturated + flooded versus fluid flooded RMSFs show significant differences between amino acids around the external linker regions between TM5 and 6, TM7 and 8 and TMs 9 and 10). There are also significant increases in the inside linker region between TM10-TM11. In the gel state, external flooding has a negligible effect on RMSF's.

(A) **(B)**

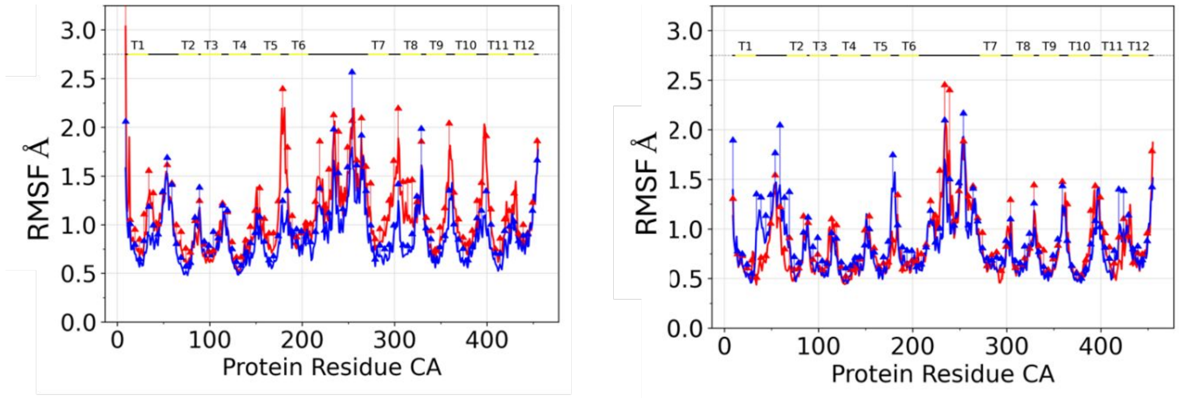

With high D-glucose concentrations in the bathing solutions ≈ 50 mM, the multiple D-glucose dependent H-bonding events agitate the external and internal extra-membranous portions of GLUT1, leading to alterations in mobility of the exposed amino acid residues [48]. The α carbon RMSFs increase in the fluid membrane state of the amino acid 190-210, corresponding to the external linker between TMs 5 and 6 and, a structured region of the long intracellular linker residues 230-260 between TMs 6 and 7 (**Figure S3**). However, saturated docking does not alter the RMSF N-terminal half of GLUT1, which is already greatly enhanced by glucose flooding, except for the internal linker between TMs 10 and 11. These

results substantiate the findings obtained from **Figure S3** that the combination of flooding + saturation of the central channel lead to a greater accessibility of GLUT1 to external ligand entry.

Figure S4. Average hydrogen bond analysis: glucose-glucose vs. glucose-water interactions. This analysis examines hydrogen bond interactions along the pore axis between pairs of glucose molecules. In each frame, a hydrogen bond is classified as replaced by either a water molecule or another glucose molecule within a 150 ps interval, with a score of 1 assigned to water or glucose, respectively. (A) Left: A decrease in the number of hydrogen bonds between two glucose molecules, coupled with an increase in water hydrogen bonds to either glucose, indicates that a water hydrogen bond has replaced a glucose-glucose hydrogen bond. Right: Conversely, a decrease in water hydrogen bonds to either glucose alongside an increase in glucose-glucose hydrogen bonds suggests that a glucose-glucose hydrogen bond has replaced a water hydrogen bond. Red labels mark pore residue locations along the Z-axis, from 33 to -33 Å. (B) Replica 1/Gel phase appears to be an outlier, as glucose molecules initially within the pore remained largely stationary, resulting in a higher density around Z=0. Excluding this replica, consistency is observed between replicas 2 and 3 in the gel phase, with less similarity to the replicas in the fluid phase where greater competition between water and glucose for hydrogen bonding leads to more dynamic interactions.

(A)

Water-Glucose replaces Glucose-Glucose H-bond interactions	Glucose-Glucose replaces Water-Glucose H-bond interactions

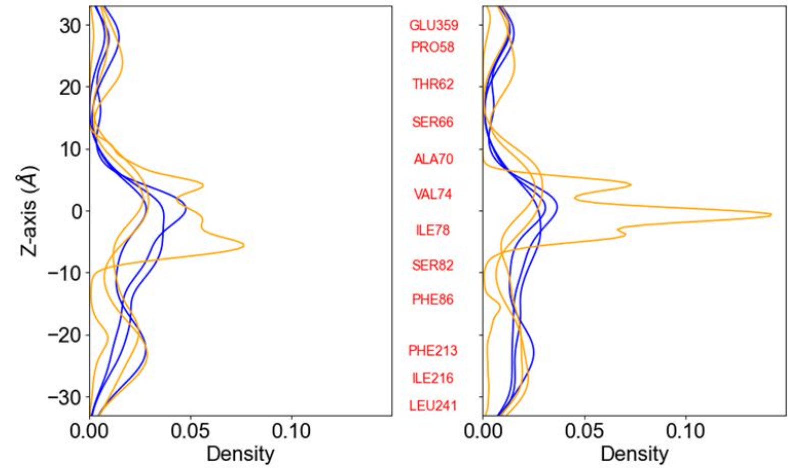

(B)

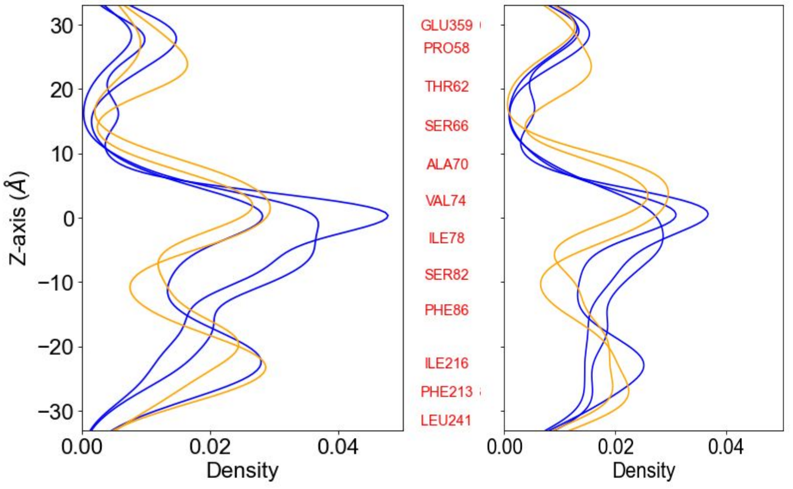

Figure S5 Hydrogen bond analysis: glucose-glucose vs. glucose-protein interactions. The density along the Z-axis indicates where a glucose molecule (BGLC) switches from hydrogen bonding with another glucose molecule to forming a hydrogen bond with a GLUT1 pore residue. Black dots mark the positions of residues that contribute the highest proportion of hydrogen bonds with any glucose molecule. Labels along the Z-axis highlight the five residues that most frequently form hydrogen bonds with new glucose molecules. The maximum time window for glucose molecules to switch hydrogen bonding to a pore residue was set to 2 ns. (A) Data represents the average across three replicas per system; (B) data represents a single replica.

(A) AVERAGE glucose-glucose vs glucose-protein H-bond interactions

Fluid Phase	Gel Phase
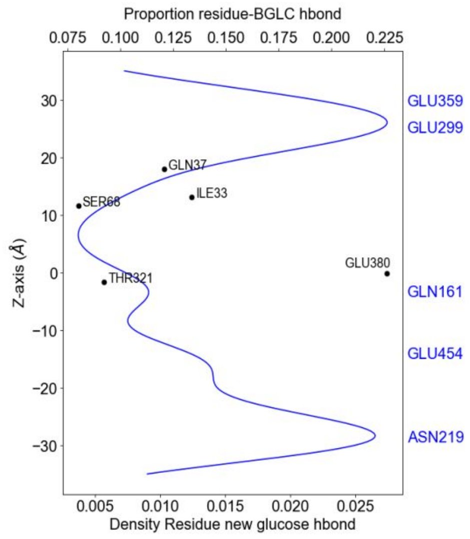	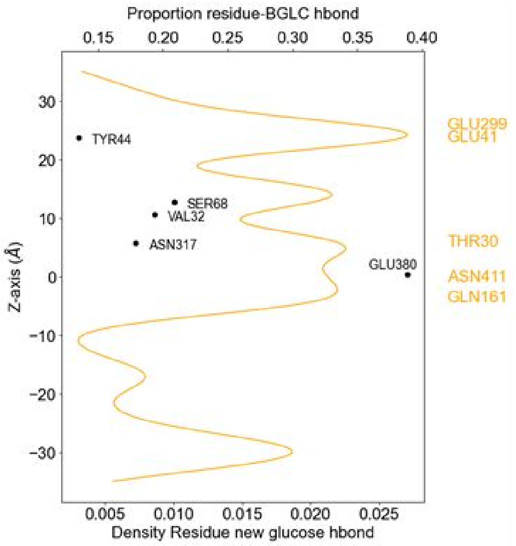

(B)

REPLICA 1

Fluid Phase	Gel Phase
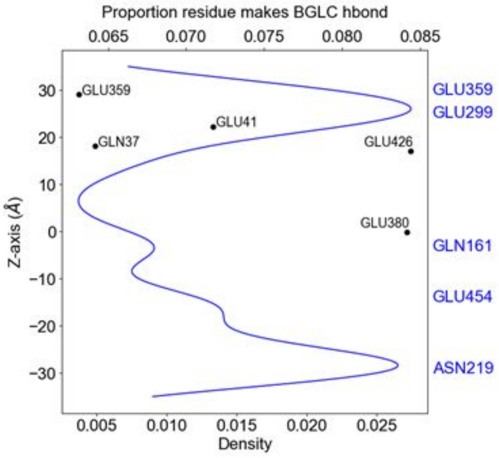	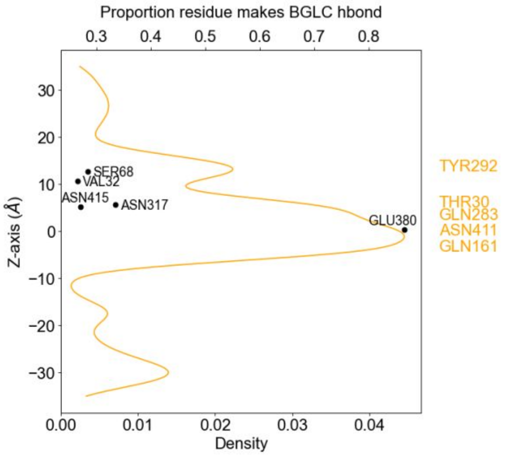

REPLICA 2

Fluid Phase	Gel Phase

<table>
<tr><td>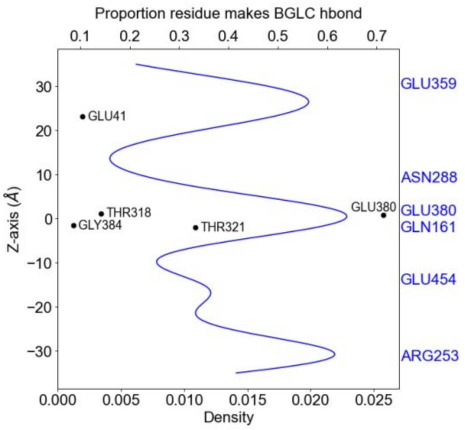
</td><td>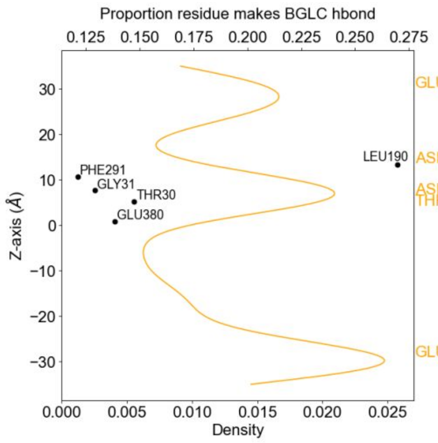
</td></tr>
<tr><td colspan="2">REPLICA 3</td></tr>
<tr><td>Fluid Phase</td><td>Gel Phase</td></tr>
<tr><td>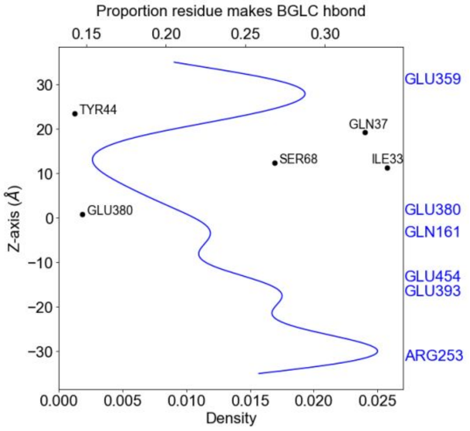
</td><td>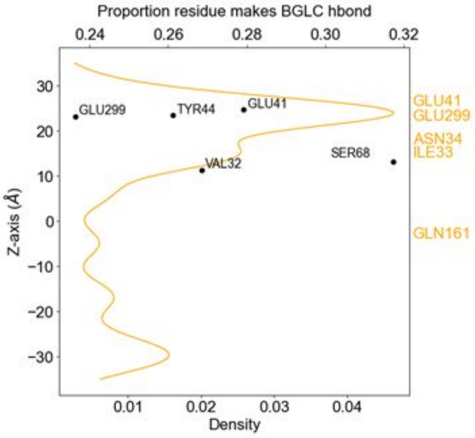
</td></tr>
</table>
